# Viral Hepatitis: Host Immune Interaction, Pathogenesis and New Therapeutic Strategies

**DOI:** 10.3390/pathogens13090766

**Published:** 2024-09-05

**Authors:** Angela Quirino, Nadia Marascio, Francesco Branda, Alessandra Ciccozzi, Chiara Romano, Chiara Locci, Ilenia Azzena, Noemi Pascale, Grazia Pavia, Giovanni Matera, Marco Casu, Daria Sanna, Marta Giovanetti, Giancarlo Ceccarelli, Pierfrancesco Alaimo di Loro, Massimo Ciccozzi, Fabio Scarpa, Antonello Maruotti

**Affiliations:** 1 Unit of Clinical Microbiology, Department of Health Sciences, “Magna Græcia” University of Catanzaro “Renato Dulbecco” Teaching Hospital, 88100 Catanzaro, Italy; quirino@unicz.it (A.Q.); nmarascio@unicz.it (N.M.); graziapavia@unicz.it (G.P.); mmatera@unicz.it (G.M.); 2 Unit of Medical Statistics and Molecular Epidemiology, Università Campus Bio-Medico di Roma, 00128 Rome, Italy; chiara.romano@unicampus.it (C.R.); m.ciccozzi@unicampus.it (M.C.); 3 Department of Biomedical Sciences, University of Sassari, 07100 Sassari, Italy; aciccozzi@uniss.it (A.C.); c.locci3@phd.uniss.it (C.L.); darsanna@uniss.it (D.S.); fscarpa@uniss.it (F.S.); 4 Department of Veterinary Medicine, University of Sassari, 07100 Sassari, Italy; iazzena@uniss.it (I.A.); npascale@uniss.it (N.P.); marcasu@uniss.it (M.C.); 5 Department of Chemical Physical Mathematical and Natural Sciences, University of Sassari, 07100 Sassari, Italy; 6 Department of Sciences and Technologies for Sustainable Development and One Health, Università Campus Bio-Medico di Roma, 00128 Rome, Italy; giovanetti.marta@gmail.com; 7 Instituto René Rachou, Fundação Oswaldo Cruz, Belo Horizonte 30190-002, MG, Brazil; 8 Climate Amplified Diseases and Epidemics (CLIMADE), Brasilia 70070-130, GO, Brazil; 9 Department of Public Health and Infectious Diseases, University Hospital Policlinico Umberto I, Sapienza University of Rome, 00161 Rome, Italy; giancarlo.ceccarelli@uniroma1.it; 10 Department GEPLI, Libera Università Maria Ss Assunta, 00193 Rome, Italy; p.alaimodiloro@lumsa.it

**Keywords:** viral hepatitis, host–pathogen interactions, immune evasion mechanisms, viral persistence, therapeutic strategies

## Abstract

Viral hepatitis is a major cause of liver illness worldwide. Despite advances in the understanding of these infections, the pathogenesis of hepatitis remains a complex process driven by intricate interactions between hepatitis viruses and host cells at the molecular level. This paper will examine in detail the dynamics of these host–pathogen interactions, highlighting the key mechanisms that regulate virus entry into the hepatocyte, their replication, evasion of immune responses, and induction of hepatocellular damage. The unique strategies employed by different hepatitis viruses, such as hepatitis B, C, D, and E viruses, to exploit metabolic and cell signaling pathways to their advantage will be discussed. At the same time, the innate and adaptive immune responses put in place by the host to counter viral infection will be analyzed. Special attention will be paid to genetic, epigenetic, and environmental factors that modulate individual susceptibility to different forms of viral hepatitis. In addition, this work will highlight the latest findings on the mechanisms of viral persistence leading to the chronic hepatitis state and the potential implications for the development of new therapeutic strategies. Fully understanding the complex host–pathogen interactions in viral hepatitis is crucial to identifying new therapeutic targets, developing more effective approaches for treatment, and shedding light on the mechanisms underlying progression to more advanced stages of liver damage.

## 1. Introduction

Despite advancements in diagnostic approaches and therapeutic strategies as well as reductions in associated costs, viral hepatitis remains the second infectious cause of mortality worldwide, contributing substantially to the burden of liver disease [1]. The World Health Organization (WHO), in the more recent “Global Hepatitis Report”, highlighted a concerning trend [1]. The estimated number of deaths, mainly due to chronic Hepatitis B (HBV) and C (HCV) infections, increased from 1.1 million in 2019 to 1.3 million in 2022, with about 3500 deaths per day [1]. Around 6000 new cases of viral hepatitis occur daily, not considering that a significant portion of the affected population is undiagnosed in resource-limited countries [1,2]. Furthermore, even among those diagnosed, the proportion receiving treatment is low [1,2]. Although this epidemiological scenario does not currently indicate the achievement of WHO’s goal to eradicate viral hepatitis by 2030 [3], it could still be attainable if immediate and decisive measures are implemented [2,4]. In this regard, leading researchers and clinicians in the field of viral hepatitis have shared their perspectives on its feasibility [2]. They discussed key areas of progress and outlined the next decade’s priorities to achieve this objective [2], among them the need for new curative therapies and therapeutic vaccines for chronic HBV [5,6,7] and the development of an HCV vaccine against infections [8,9]. These efforts aim to mitigate chronic disease progression, cirrhosis, liver cancer, and mortality rates.

Despite significant advances in the understanding of viral hepatitis infections, multiple gaps remain in the literature, particularly in relation to the intricate mechanisms governing host–pathogen interaction and immune evasion, as well as in the understanding of viral persistence and its implications for the development of new therapeutic strategies. Although numerous studies have addressed individual aspects of these processes, a critical synthesis that integrates these elements to provide a comprehensive and up-to-date view of therapeutic challenges and opportunities is lacking. This review aims to fill these gaps, offering an integrated analysis of the most recent scientific findings and highlighting mechanisms that have not been fully explored and that may prove crucial for improving therapeutic approaches and preventing progression to more advanced forms of liver damage. Specifically, this work aims to provide a comprehensive examination of the dynamics of host–pathogen interactions in viral hepatitis, the key mechanisms regulating viral entry, replication, immune evasion, hepatocellular damage, and viral persistence, and their implications for developing new therapeutic strategies (Table 1).

Fully understanding these complex interactions is essential for identifying novel therapeutic targets, improving treatment approaches, and shedding light on the mechanisms underlying the progression to more advanced liver disease stages.

## 2. Overview of Viral Hepatitis and Host–Pathogen Interactions

Viral hepatitis encompasses a group of viruses that primarily affect the hepatic cells, leading to varying degrees of liver inflammation, injury, and dysfunction [10,11]. The host–pathogen interactions in viral hepatitis are pivotal in determining disease progression and outcomes across the spectrum of hepatitis viruses [12]. The clinical manifestations can range from asymptomatic to acute or persistent/chronic infections, which lead to several liver-related complications, including cirrhosis and hepatocellular carcinoma (HCC) [10,11]. Five primary hepatotropic viruses are responsible for hepatitis, Hepatitis A virus (HAV), HBV, HCV, Hepatitis D virus (HDV), and Hepatitis E virus (HEV), each exhibiting unique virological and pathogenic characteristics [10,11]. Less frequently, viral hepatitis can be caused by non-hepatotropic viruses like Cytomegalovirus (CMV), Epstein–Barr virus (EBV), Herpes simplex virus (HSV), and Varicella-zoster virus (VZV) [13,14], which generally do not target the liver and rarely induce hepatitis in immunocompetent individuals [14]. The epidemiological, virological and clinical aspects of primary hepatotropic viruses are summarized in Table 2.

### 2.1. Hepatitis A Virus

HAV is a quasi-enveloped, single-stranded (ss) RNA virus belonging to the *Picornaviridae* family, genus *Hepatovirus* [16,17]. It was first discovered in 1973 using immunoelectron microscopy [18]. HAV infection is transmitted by fecal-oral route [19] and annually leads to over 150 million new cases worldwide [1]. From 2021 to 2022, there was nearly a 60% decrease in newly reported HAV cases due to improved sanitation procedures and the widespread use of an effective vaccine [20,21]. The HAV genome (7.5 kb) encodes a single polyprotein, subsequently cleaved into structural proteins (VP4, VP2, VP3, and VP1pX capsid antigens) and non-structural proteins, such as 2B, 2C, 3A, 3B (the genome-linked protein, VPg), 3Cpro (a cysteine protease), and 3Dpol (RNA-dependent RNA polymerase), which are essential for viral replication and assembly [16,22]. The significant variability in HAV RNA genomic sequences [23] has led to the identification of seven genotypes (I-VII). Four of these (I, II, III, and VII) infect humans, while genotypes IV, V, and VI infect simians [24]. Clinical manifestations vary from asymptomatic cases to acute hepatitis characterized by jaundice and elevated liver enzymes [22,25]. Unlike other hepatotropic viruses, HAV typically induces an acute inflammatory response in the liver that generally resolves without leading to chronic conditions, followed by life-long immunity [26]. Nevertheless, host factors such as age, pregnancy, immune status, and pre-existing liver conditions may contribute to a prolonged or recurring illness in up to 20% of individuals, and <1% may develop acute liver failure [26]. Since no chronic infection is known, natural or vaccine-acquired immunity persists lifelong, and there is a low probability of liver failure; the virological and immunological mechanisms of acute self-limited HAV infection will not be addressed in the following paragraphs [22,25,26].

### 2.2. Hepatitis B Virus

HBV is an enveloped DNA virus belonging to the *Hepadnaviridae* family, genus *Orthohepadnavirus*. The first discovery was in the 1960s when Baruch S. Blumberg identified the “Australia antigen” (HBV surface antigen, HBsAg) in the blood of an Australian Aboriginal patient [27]. Overall, it is estimated that around 350 million people worldwide are living with chronic infection [1]. The transmission occurs through contact with infected blood or bodily fluids and by vertical transmission at birth, a significant route of spread in endemic regions [28,29]. The viral genome (3.2 kb) is constituted by partially double-stranded relaxed circular DNA (rcDNA), replicating through an RNA intermediate [14]. It encodes several key proteins: the viral DNA polymerase (reverse transcriptase), the hepatitis B x protein (HBxAg), and three viral antigens: hepatitis B core antigen (HBcAg), HBsAg, and hepatitis B e antigen (HBeAg), a secretory protein processed from the pre-core protein [14]. HBV is classified into ten genotypes (A to J) and numerous sub-genotypes based on genetic divergence due to reverse transcriptase lacking proofreading activity [30]. HBV genotypes significantly impact clinical outcomes, including the rate of HBeAg seroconversion, liver disease severity, the emergence of viral mutants, transmission patterns, and the effectiveness of therapy [31,32,33,34,35,36]. The majority of individuals who contract the virus in adulthood develop an acute self-limited infection [37]. Less than 5% of immunocompetent adults exposed to HBV develop a persistent infection, known as chronic HBV [28,29]. In contrast, HBV infections contracted during infancy or early childhood progress to chronicity [28,29]. The integration of viral DNA into the host genome as covalently closed circular DNA (cccDNA) during chronic infection, contributes to the persistence of HBV and increases the risk of severe liver diseases such as liver failure, cirrhosis, and HCC [37,38]. Preventive measures, such as a prophylactic vaccine, are crucial for controlling HBV infection [39]. However, it is ineffective for patients who are already chronically infected. In these cases, current treatments are limited to immunomodulatory agents, such as conventional or pegylated type I interferons (pegIFN-I), or a few direct-acting antivirals (DAAs), known as third-generation nucleos(t)ide analogues (NUCs), which target the viral reverse transcriptase [39]. Similarly to anti-HIV drugs, NUCs rarely achieve complete viral eradication from the liver, necessitating lifelong treatment for most patients, which induces drug resistance [40].

### 2.3. Hepatitis D Virus

HDV is a member of the *Deltaviridae* family and genus *Deltavirus*, identified in the mid-1970s in Italy from HBsAg-positive patients with chronic liver disease [41]. Approximately 15 to 20 million people are estimated to be infected worldwide [1]. It is the smallest known human virus, with a circular ss-RNA genome (1.7 kb), encoding a single protein. The HDAg protein is expressed in two isoforms: large (L-HDAg) and small (S-HDAg), which share identical amino acid sequences except for an additional nineteen residues at the C-terminus of the L form, leading to distinct biological functions. S-HDAg is involved in viral replication, whereas L-HDAg inhibits replication and facilitates the packaging of mature virions through interactions with specific host cell enzymes [42,43]. As a satellite virus, it requires co-infection (simultaneous infection with HBV and HDV) or superinfection (HDV infection in a person with chronic HBV) for its propagation within humans, leading to the most severe form of viral hepatitis [44]. The virus utilizes HBsAg to facilitate the release of its progeny and to mediate de novo entry into hepatocytes [44]. Mirroring the transmission pathway of HBV, it is predominantly transmitted through parenteral exposure to contaminated body fluids, intravenous drug use, and vertical transmission (infrequent occurrence) [44,45,46]. HDV RNA genome exhibits significant heterogeneity, comprising eight distinct genotypes which display variations in disease outcomes and treatment responses [47]. HDV genotype 1 is the most prevalent worldwide, associated with a more severe liver disease, and is primarily found in Europe and North America. HDV genotype 2 is mainly present in Asia, while genotype 3 is highly pathogenic and has been responsible for fulminant HDV outbreaks in the Amazon basin. HDV genotype 4 is prevalent in Japan and Taiwan, while genotypes 5 through 8 are predominantly seen in Africa [48,49]. Co-infection or superinfection with HBV and HDV leads to rapid progression of cirrhosis in 15% of cases (within 1–2 years) and in 70–80% of cases (within 5–10 years). Furthermore, the incidence of HCC and hepatic decompensation is 2–3 times higher in patients with HBV-HDV co-infection compared to those with HBV mono-infection [50,51]. The HDV infection can be prevented by HBV immunization, but this does not protect those already infected with HBV. Pegylated interferon alpha (pegIFNα) is the standard treatment for HDV infection, typically prescribed for at least 48 weeks to control liver disease progression. Despite its effectiveness, it can cause substantial side effects and is not recommended for people living with decompensated cirrhosis, active psychiatric disorders, or autoimmune conditions. Bulevirtide (BLV), a newer treatment option, shows promise as an entry inhibitor that blocks HDV entry in liver cells. In July 2020, the European Medicines Agency (EMA) granted BLV conditional marketing authorization for chronic HDV infection, advising that the treatment be continued as long as clinical benefits are observed [52,53].

### 2.4. Hepatitis C Virus

HCV is an enveloped virus (positive ss-stranded RNA genome, 9.6 kb), belonging to the *Flaviviridae* family, genus *Hepacivirus*, affecting around 58 million people worldwide [1]. It was first identified in 1975 by Feinstone S.M. and colleagues when many cases of transfusion-associated hepatitis were not linked to HAV or HBV [54]. In 1989, Houghton M. and colleagues, in collaboration with the Centers for Disease Control and Prevention (CDC), successfully isolated, cloned, and sequenced the HCV genome from high-titer samples obtained from an experimentally infected chimpanzee [55,56], leading to significant advancements in diagnosis and treatment. The transmission routes include intravenous drug use, blood-to-blood contact, unsafe medical practices, transfusion of unscreened blood, and vertical transmission [57,58]. The polyprotein is cleaved by viral and host proteases into three structural proteins (Core, E1, and E2) and seven non-structural proteins (p7, NS2, NS3, NS4A, NS4B, NS5A, and NS5B) [59]. HCV exhibits significant genetic diversity, leading to the progressive diversification and global dissemination of its genotypes and subtypes [60]. Genotypes differ by approximately 30%–35% of their nucleotide sequence, while subtypes vary by more than 15% [60]. Currently, 8 different genotypes and over 100 subtypes have been reported, with the possibility of additional variants [60]. However, a limited HCV subtypes number, such as genotypes 1a, 1b, 2a, 2b, 2c, 3a, 4a, 5a, and 6a, predominate among infected individuals in industrialized nations [61]. Its high genetic variability, particularly in the hypervariable regions of the E2 glycoprotein, enables the “quasispecies” virus to evade the host immune response and establish chronic infection [62]. Chronic HCV infection can lead to liver fibrosis, cirrhosis, and HCC. Despite the availability of highly effective direct-acting antivirals (DAAs) that can achieve sustained virological response (SVR) in more than 95% of patients [63], the high HCV genetic plasticity could enable the virus to develop quickly antiviral resistance mutations [64,65,66,67].

### 2.5. Hepatitis E Virus

HEV is a quasi-enveloped, positive-sense ss-RNA virus classified within the genus *Orthohepevirus* of the *Hepeviridae* family, causing approximately 20 million cases annually, primarily in developing countries [1,68,69]. It was first discovered in the 1980s, during the Soviet occupation of Afghanistan. The researcher Balayan M.S., following an unexplained hepatitis outbreak at a military camp, ingested a pooled fecal extract from affected soldiers and subsequently identified the new virus, designed as HEV, in his stool samples using electron microscopy [70]. The HEV genome (~7.2 kb) encodes three open reading frames (ORFs) [71,72]. The ORF1 polyprotein encodes the non-structural proteins and includes several functional domains: a methyltransferase (MeT), a putative papain-like cysteine protease (PCP), a hypervariable region (HVR), an RNA helicase (Hel), and an RNA-dependent RNA polymerase (RdRp). The ORF2 genomic region encodes the capsid protein, while ORF3 produces a multifunctional protein essential for virion morphogenesis and release, as well as to mediate various interactions with host cell components [68,73,74,75,76]. The virus encompasses eight distinct genotypes, of which HEV-1, HEV-2, HEV-3, HEV-4, and HEV-7 are known to infect humans [77,78]. HEV-1 and HEV-2 are endemic to developing regions and are primarily transmitted through the fecal-oral route via contaminated water [73]. In contrast, HEV-3, HEV-4, and HEV-7 genotypes are mainly harbored in pigs, boars, deer, and dromedary camels (genotype 7), which can infect humans, leading to zoonotic transmission [77,78,79]. HEV infection typically results in acute, self-limiting hepatitis, with symptoms including jaundice, fever, fatigue, and abdominal pain [80]. However, chronic HEV infection can occur in immunocompromised individuals, including organ transplant recipients, those undergoing immunosuppressive therapy, as well as patients with preexisting liver disease [81,82,83,84,85], leading to progressive hepatic disease and cirrhosis [85]. Furthermore, in pregnant women, HEV infection is linked to a broad range of adverse outcomes for both the mother and fetus. Recently, Liang Y. and colleagues [86] reported a significant association between decreased immune responses during HEV infection and fulminant hepatitis in pregnant women. This includes impaired T-cell activation, leukocyte cell–cell adhesion, regulation of lymphocyte activation, and immune response-regulating signaling pathways [86]. Currently, the recombinant HEV 239 vaccine (Hecolin), based on HEV-1, is the only globally available vaccine for HEV. In October 2012, the Chinese Food and Drug Administration (CFDA) approved the first HEV vaccine [87,88,89]. The phase 3 trial demonstrated that the vaccine prevented HEV with 100.0% efficacy (95% CI 72.1–100.0) within 12 months after the third dose [90]. Additionally, a recent evaluation of the vaccine’s long-term efficacy, based on a 10-year follow-up of the phase 3 trial, showed that the three-dose regimen provided 86.6% efficacy over 10 years. The vaccine induces antibodies lasting for at least 8.5 years [91].

## 3. Mechanisms of Viral Entry

The entry of primary hepatotropic viruses into hepatic cells involves distinct molecular mechanisms tailored to each virus’s unique structure and replication strategy. Understanding the detailed mechanisms of hepatitis virion entry is crucial for designing targeted interventions and advancing knowledge of liver pathogenesis during acute or chronic hepatitis.

### 3.1. Mechanisms of Viral Entry: Hepatitis B and Hepatitis D Viruses

Circulating HBV virions, known as “Dane particles”, are enveloped by HBsAg, consisting of three virus-coded surface proteins, large (L), middle (M), and small (S) [92]. The inner nucleocapsid is formed by 120 HBcAg homodimers, containing the rcDNA. Approximately 10% of the viral particles encompass double-stranded linear DNA (dslDNA), and around 1% carry primarily unspliced forms of HBV RNA [93]. HBV virion binds the surface of hepatocytes through interactions with heparan sulfate proteoglycans (HSPGs), albeit with low affinity. The subsequent binding occurs with the sodium taurocholate co-transporting polypeptide (NTCP) receptor, which interacts with the pre-S1 domain of L-HBsAg, with high affinity. This interaction may also prompt the internalization of HBV into hepatic cells via endocytosis [29]. Furthermore, the epidermal growth factor receptor (EGFR) has been shown to associate with NTCP, facilitating HBV entry into cells [94]. After the entry of the virus into the hepatic cell, the viral envelope merges with the endosomal membrane, releasing the nucleocapsid into the cytoplasm. The nucleocapsid then uses the microtubule network and associated motor proteins to move toward the nucleus [95]. Within the nucleus, the rcDNA of HBV is converted into cccDNA, and some HBV DNA may integrate into the host genome, a key factor in the persistence of HBV infection [29]. Similar to HBV, HDV also uses the NTCP receptor for entry and is internalized into the hepatocyte through clathrin-mediated endocytosis [96].

### 3.2. Mechanisms of Viral Entry: Hepatitis C Virus

HCV interacts with hepatocytes through a complex, multistep process that involves various host-cellular factors utilized by the viral envelope glycoproteins E1 and E2 [97]. These glycoproteins are responsible for targeting the cell, initiating endocytosis, and facilitating membrane fusion, which is triggered by the acidification of endosomes [98]. Four key cellular factors are crucial for HCV’s attachment and internalization: CD81, scavenger receptor class B type I (SR-BI), claudin-1 (CLDN), and occludin (OCLN). Among these, the interaction between E2 and CD81 is the main target for antibody-mediated neutralization [99]. While CD81 is widely expressed in various cell types, suggesting its role extends beyond hepatocyte-specific binding, it relocates with the virus to tight junctions and interacts with CLDN and OCLN in the endosome, facilitating the necessary acidification for viral entry. CD81 is a tetraspanin family membrane protein with four transmembrane domains. Its extracellular loop, known as CD81-LEL, consists of a globular domain with five helices (A–E) that binds to E2, with key interacting residues previously identified [100,101,102,103,104,105]. However, the precise molecular mechanisms involved in HCV’s entry and membrane fusion remain unclear [106].

### 3.3. Mechanisms of Viral Entry: Hepatitis E Virus

Despite evidence suggesting HEV has caused outbreaks since ancient times, our understanding of its entry mechanisms into hepatic cells, replication cycle, and interactions with host cells has only recently advanced [107]. HEV was first defined as a “naked” virion (nHEV); subsequently, it has been shown that new virions are released in the bloodstream (leading viremia) and in the biliary tract from the liver with an outer lipid layer [108,109]. The high concentration of bile acids (present in proximal bile canaliculus) removes the membrane from “quasi-enveloped” virions (eHEV), resulting in the fecal shedding of nHEV [107]. The eHEV are infectious but lack virus-encoded proteins on their surface, setting them apart from conventional enveloped viruses [107]. Several observations are consistent in indicating that the biogenesis of eHEV viral particles involves highly specific sorting of viral capsids into multivesicular endosomes (MVEs) in endosomal sorting complexes required for transport (ESCRT)-dependent process, mirroring the production of exosomes [110]. The ORF3 palmitoylated protein, associated exclusively with eHEV virions and not with nHEV virions, recruits TSG101, a component of ESCRT-I, facilitating its association with the cytosolic leaflet of membranes, which is essential for eHEV release [111,112,113]. As previously reported, HEV virions, releasing from polarized hepatocytes, showed co-localization of ORF3 and ORF2 proteins in vesicular structures [114,115]. In a polarized hepatocyte culture and a human liver chimeric mouse models, Sari G. and colleagues showed that the absence of ORF3 significantly reduced HEV replication and the release of virions from the apical surface, but not from the basolateral surface, of polarized hepatocytes. Moreover, whereas wild-type HEV was able to establish a persistent infection in humanized mice, the HEV ORF3-null mutant, despite exhibiting transient replication in the liver, was unable to sustain the infection and was subsequently cleared [115]. The viral entry of enveloped viruses typically occurs via viral peplomers, involving membrane fusion [116]. However, such proteins are absent in eHEV. The initial attachment and endocytosis of eHEV virions likely involve non-specific interactions with clathrin, and similar to nHEV, they undergo GTPase Ras-related protein Rab-5A (RAB5A) and RAB7A-dependent trafficking to endolysosomes [117]. The potential roles of phosphatidylserine receptors and integrins in eHEV entry remain undefined [107]. The eHEV membrane lipids degradation is mediated within lysosomes by cellular enzymes [118]. In endolysosomes, the eHEV membrane is degraded by lysosomal acid lipase (LAL) and Niemann–Pick C1 protein (NPC1), aiding in the breakdown of eHEV [117,119]. Lysosomal proteases may also play a role in the entry of quasi-enveloped viruses by degrading the ORF3 protein, although it is unclear whether this action is required for subsequent entry steps [107]. Despite reports that ORF3 showed ion channel activity, this function is unlikely to be significant for viral entry [107]. The compartment and mechanism of eHEV capsid uncoating remain unknown. The endolysosome may function merely as a transitional compartment where the virus sheds its quasi-envelope. Overexpressed ORF2 proteins are known to translocate to the cytoplasm through the endoplasmic reticulum-associated degradation pathway [120]. As of the entry mechanisms of nHEV particles, limited information is available. In some cases, viruses without envelopes enter host cells through interactions between their capsid proteins and receptor molecules on the cell surface, which trigger the uncoating of the viral genome and endocytosis [121]. A portion of the ORF2 protein was identified as a potential receptor-binding site due to polysaccharide-binding activity observed in recombinant HEV-like particles [122,123], but no specific receptor molecule has yet been reported. The entry of nHEV appears to be at least partially reliant on clathrin- and dynamin-dependent endocytosis, though it does not involve RAB5A or RAB7A [124]. This suggests that the uncoating of nHEV likely begins early within the endocytic pathway [107].

## 4. Host Immune Response

The host immune response, including innate and adaptive immunity, protects against pathogens, although dysregulation or over-activation could cause illness. The innate immune system is the first line of host defense and rapidly contrasts viral infections [125]. Viruses’ recognition occurs through several receptors able to sense molecular structures of these pathogens, such as pathogen-associated molecular patterns (PAMPs) and pathogen recognition receptors (PRRs). The innate signaling receptors consist of the Toll-like receptors (TLRs), the main elements of the hepatic immune system, a retinoic acid-inducible gene I (RIG-I)-like RNA helicases (RLHs), melanoma differentiation-associated 5 (MDA-5), and nucleotide-binding oligomerization domain (NOD)-like receptors (NLRs) [126]. The major components of antiviral innate immune response (IR) are Type I interferons (IFNα and IFNβ) and type III IFNs (IFNλ), binding IFNα/β (IFNAR1–IFNAR2) and IFNλ receptors (IFNLR1–IL10R2), respectively [127]. Both IFN proteins induce the expression of IFN-stimulated genes (ISGs) mediated by ISGF3 complex, consisting of IFN regulatory factor 9 (IRF9), phosphorylated signal transducer and activator of transcription 1 (STAT1), and phosphorylated [128]. Pro-inflammatory chemokines and cytokines induce an inflammatory response and enroll several immune cells [125]. In liver tissue, the innate immune system leads a robust response through natural killer (NK), Kupffer (KCs), and hepatic dendritic (DCs) cells [129]. On the other hand, the adaptive IR induced by T cells (CD4+ and/or CD8+) and B cells (producing virus-specific antibodies) is mainly involved in viral hepatitis spontaneous resolution (acute infection) or persistence (chronic infection) [130]. The quality and quantity of patient IR essentially determine the progress of infection and the clinical outcome [125].

### 4.1. Innate and Adaptive Immune Response: Hepatitis B and Hepatitis D Viruses

The interactions between HBV and the immune system are crucial for the virus’s persistence. However, the specific viral and host factors that determine whether an acute infection is cleared or progresses to chronic disease remain poorly understood. Innate immunity is essential in the initial response to viral infections and supports the activation of adaptive immunity [131]. HBV establishes infection without being detected by the innate immune system, avoiding the activation of antiviral pathways (failure to induce IFN in hepatocytes), although IFN can still reduce viral replication [131]. HBV is considered a furtive virus because it does not trigger a strong innate immune response. Instead, it disrupts extracellular matrix–receptor interactions and oxidative phosphorylation, creating an environment that supports its persistence [132]. The loss of HBsAg or achieving a functional cure is regarded as the optimal therapeutic outcome for patients with chronic infection. Existing antiviral therapies can suppress viral replication but do not eliminate the infection because of the persistent cccDNA in hepatocytes [133,134]. The viral infection clearance depends on a strong and sustained adaptive immune response. Virus-specific CD8+ T cells play a key role in resolving acute HBV infection [28]. Nevertheless, in patients with chronic hepatitis B, HBV-specific CD8+ T cells become terminally exhausted due to prolonged exposure to high levels of viral antigens [135]. Recent studies showed that the compromise of adaptive immune responses is influenced not only by the high antigen load but also by the duration of exposure to viral antigens [136,137]. The innate immune response is functionally impaired in chronic HBV infection. Zimmer et al. found a positive correlation between the increased cytotoxic activity of NK cells and liver inflammation, particularly in patients who clear HBsAg after stopping NUCs treatment [138].

In liver, the CD4+ T cells may enhance innate immune activation by promoting the priming and proliferation of NK cells. IL-2, produced by a small number of T cells, might be involved in this priming process. The notable increase in HBV-specific CD4+ T cells observed in resolved chronic infection may help compensate for the inadequate immune response caused by dysfunctional HBV-specific CD8+ T cells, many of which are likely exhausted due to chronic exposure to pseudo-antigens produced by HBV, such as HBeAg and various splice isoforms of HBsAg [139]. HBV does elicit an early innate immune response characterized by FN-γ, IL-12, p70, and IL-17A production. At baseline, these cytokines were undetectable but peaked early and then rapidly declined before the peak of ALT levels and the onset of viral clearance. The early detection of IFN-γ within the first 3 weeks in infected chimpanzees, followed by its rapid decrease, may account for the inconsistent findings reported in the literature regarding HBV-induced innate responses, particularly in human studies where the timing of HBV infection is often difficult to ascertain [140]. During the first phase of infection, NK and NKT cells, which represent the primary intrahepatic lymphocytes (30% to 40%), are likely a main source of cytokine release. A comparison of cytokine profiles between severe and classic acute hepatitis B caused by an HBV pre-core mutant revealed that the extent of liver damage in severe AHB was associated with a distinct cytokine profile. This profile was characterized by minimal levels of IFN-γ, IFN-α, IL-12 p70, and IL-17A throughout the disease course. Notably, this muted cytokine profile was linked to a limited inflammatory infiltrate in the liver and reduced activation of caspase 3, a marker of hepatocyte apoptosis [140]. Furthermore, Li et al. showed that in B-cell hyperactivation during chronic HBV progression, TLR4 played a key role in inducing downstream activation of MyD88/NF-κB in HBV-triggered B cells [141].

HBV and HDV co-infection or superinfection pose a significant health burden due to their potential to cause severe liver disease, including fulminant hepatitis, cirrhosis, and hepatocellular carcinoma (HCC) [142]. HBV/HDV infection is characterized by dynamic patterns of HBV and HDV dominance that fluctuate over time. Therefore, it is essential to investigate both HBV-specific and HDV-specific immune responses. Research using mouse models of HDV infection, as well as studies in patients, demonstrated that innate immune responses mediated by monocytes and NK cells play a role in accelerating liver damage in co-infection [142]. Moreover, in an immunodeficient humanized mouse model of HBV/HDV infection, co-infected mice exhibited a heightened inflammatory response with elevated levels of both basal pro-inflammatory and pro-fibrogenic cytokines. This increased cytokine activity was associated with greater liver damage compared to uninfected mice and those infected with HBV alone [143]. Previous immunological studies in chronic HBV infection revealed increased HBcAg/HBeAg-specific T cell proliferation, both before and during ALT flares, accompanied by elevated production of Th1 cytokines, including IFN-γ and IL-2 [144,145]. Joshi et al. found that the Th1 response was barely detectable in HDV-RNA positive cases despite the presence of ALT flares, indicating that T-cell responses in HBV/HDV co-infection are regulated differently than in HBV mono-infection [142]. In HDV co-infected patients who exhibit viremia, there is increased TNF-alpha release from monocytes, which is linked to weaker HBV and HDV-specific T-cell responses compared to HDV-RNA negative patients, irrespective of antiviral treatment status [142]. In a recent study, the IFN-γ and IL-12 p70 levels were similar between chronic patients and the control group, while IL-10, IL-17A, TNF-α, and CXCL9 were elevated in chronic patients [146]. In vitro stimulation of CD4+ and CD8+ T cells from chronic patients displayed low levels of IFN-γ and TNF-α [144]. HBV and HDV are not hidden from the innate immune system [147]. HBV-HDV coinfection elicits a strong innate immune response. MDA5, a cytoplasmic RNA sensor, recognizes HDV RNA replication and activates innate immunity. However, despite this activation, innate immunity appears to inhibit HBV replication, whereas it does not impair HDV replication. Therefore, it has been suggested that HDV adapted to the IFN-activated state in the liver by blocking or escaping the IFN system [148]. Some innate pathways support the replication of HDV productivity; in fact, IFN induces the RNA-editing enzyme adenosine deaminase (ADAR), which is required for RNA editing, enabling viral morphogenesis. In addition, IFN production and MDA-5-mediated ISG induction also contribute to a cytokine environment that recruits professional antigen-presenting cells, thus enabling the priming of functional T cells [149].

HDV infection induces the IFN system activation in human hepatocytes, determining HBV suppression. Both hepatic viruses probably have a different sensitivity to IFN, and during superinfection, HDV outcompetes HBV, leading to a viral interference [150]. At present, the role of HDVAg in eliciting adaptive immune response is controversial. In chronic HDV infection, the specific circulating CD4+ and CD8+ T cells are rather low, with a predominance of TH1 cells mainly targeting the N-terminal region of HDAg epitopes [149]. As during HBV and HCV chronicity, the HDV-specific CD8+ T cells frequency is very low [151,152]. CD8+ T cell function is hampered if viral infection is persistent, which results in increased inflammation and constant antigen exposure [149].

### 4.2. Innate and Adaptive Immune Response: Hepatitis C Virus

The acute HCV infection in the early phase (high viral load) and ISGs high levels were probably associated with viral eradication failure. On the other hand, the activated NK cells confer an important immunoregulatory role, blocking the development of acute infection. In particular, natural killer group 2 member D (NKG2D+) cells increased IFNγ production and cytolytic activity [130]. During the innate response, infected hepatocytes can release IFNs and PAMPs, activating TLR1/2, RIG-1 and MDA5 pathways in macrophages and dendritic cells, which in turn produces IFNα, IFNβ, and IFNλ via the NF-kB pathway [153]. MDA5 could recognize the virus to trigger the host’s innate immune response during HCV infection and it is also the main component inducing IFN production. At the same time, HCV can activate RIG-1, suggesting that both pathways could be related to the progression of infection [129]. In contrast to innate, the adaptive immune response is activated several weeks after HCV infection [154]. The role of humoral response in the eradication process is still unclear; infected individuals developed neutralizing antibodies (nAbs) against HCV proteins without efficacy on viral replication. Even patients able to resolve acute infection displayed higher nAbs titers than chronic disease patients, who developed them in a delayed manner [155,156]. In most patients, B cells failed to produce a high quantity of nAbs, probably due to their intrinsic mechanisms as well as to the reduction or dysregulation from CD4+ T-cells [157]. B cell dysregulation, producing autoantibodies, such as anti-immunoglobulin autoantibody rheumatoid factor, was also related to extrahepatic diseases (i.e., mixed cryoglobulinemia) [158]. In the primate study, viral antigens were detected in peripheral and intrahepatic CD4+ T-cell responses, but only the intrahepatic response was associated with viral clearance. In peripheral CD8+ T-cells, response was detected after stimulation using different antigens. Interestingly, full or partial viral eradication showed the activation of CD8+ T-cells, producing IFNγ, in the early phase of HCV acute infection [154]. In the same experiment, during reinfection, the eradication was accompanied by both intrahepatic CD8 T-mediated cytolytic activity and the expansion of peripheral memory T-cell (CD4+ and CD8+) response. Additionally, the role of intrahepatic CD8+ T cells was confirmed in long-term protection [154]. HCV clearance by an effective IR decreases the number of CD8+ T-cells expressing high levels of CD127, which rapidly re-expand during reinfection [157]. Unlike animal models, the activation of CD38− IFNγ+ CD8+ T-cells by the CD4+ T-cells expansion was successfully related to HCV clearance in humans [154]. CD4+ T-cell response is maintained in acute-resolving infection, while it is deleted in acute-persistent infection; finally, CD4+ T cells were not detected once the chronic infection was established [157]. Chronic HCV patients showed inadequate T-cell response (virus-specific CD8+ T-cells) and the malfunction of NK cells, both incapable of secreting IFNγ. In NK cells, lacking NKp46 and NKp30 surface repertoire, lower expression of natural cytotoxicity receptor (NCR) and higher levels of NKG2A were associated with viral persistence [130]. Disease progression was related to crosstalk among cells of the diseased microenvironment, including macrophages, which released the inflammatory cytokines (IL6 and IL-1β) [159]. In cultures with infected hepatocytes, macrophages released CCL5, activating different fibrogenic and inflammatory markers’ expression [160]. Additionally, the prolonged exposure to HCV antigens exhausts T cells, upregulating inhibitory receptors, such as PD1 (programmed cell death protein 1), CTLA4 (cytotoxic T lymphocyte antigen 4), TIM3, KLRG1 (killer cell lectin-like receptor G1), CD160, and 2B4 (also known as CD244), in half of chronic patients [156]. During chronic infection, Treg cells’ (CD4+ CD25+) number increased in the liver and blood, in this last correlated to viral load, indicating that Treg cells contribute to persistence via virus-specific effector T cells suppression [156]. The CD4+ T-cells were impaired in positive patients compared to patients resolving the infection; in addition, low levels of IFNγ and IL2 were detected in chronic patients [161]. The Treg-cells number was inversely related to the CD4+ and CD8+ T-cells functionality. In HCV non-resolvers’ subjects, CD8+ T-cells proliferation and function were suppressed, while CD4+ CD25+ T-cells were expanded [162]. Humoral IR to viral proteins appears one/two months after acute infection without effective protection or infection control; HCV replication is more rapid than B cell responses. During chronic infection, the persistent activation of B-cells provided extrahepatic manifestations, such as cryoglobulinemia, and increased the atypical memory B-cell frequency due to continuous viral antigenic stimulation. However, the cells’ function is not completely abolished since they maintain the ability to be activated in vitro after the HCV antigens stimulation [163].

### 4.3. Innate and Adaptive Immune Response: Hepatitis E Virus

The activation of TLR signaling produced IL-8, which recruits dendritic cells, neutrophils, and macrophages, inducing phagocytosis and cell death. HEV infection significantly upregulated IL-8 promoter activity, displaying an antagonistic effect to IFN-β and IFN-γ, and activated the activator protein 1 (AP-1) binding the promoter and inducing IFN-β enhanceosome [164,165]. TLR7/8 recognized ssRNA, activating the innate antiviral and inflammatory responses. The two distinct pathways TRIF adaptor or MyD88 molecules can promote TLR signaling. In the presence of MyD88 deficiency, the induction of CCL5 and IL-6 was attenuated. On the contrary, TRIF knockdown does not affect IL-6 levels. In pregnant women who developed acute liver failure, the expression levels of IFN-γ were higher, while downregulation of TLR7 was observed [125]. The IFN signaling network is regulated by IRF1, its overexpression inhibits viral replication [166]. HEV induces a sustained IFNλ response by both RIG-I and MDA5, not sufficient to eliminate the virus. The viral genome mainly activated RIG-I PAMP, inducing an IFN response specific to infected host cells; additionally, the RIG-I ectopic overexpression promoted the antiviral ISGs transcription, establishing an anti-HEV status [129]. Interestingly, MDA5 can inhibit viral replication without IFN production, partially dependent on the JAK-STAT cascade [125]. In infected lung epithelial cells, the RIG-I transcription level increased 48/72 h after infection, while the MDA5 level did not change, and several inflammatory chemokines and cytokines, such as IL-6, IL-8, and TNF-α, were upregulated. Antiviral IR and viral clearance are crucially mediated by RIG-I according to in vitro experiments [166]. HEV is responsible for inflammation-mediated liver disease, while acute viral hepatitis and fulminant hepatic failure showed a difference in the cytokine profile produced by Th1 and Th2 cells. The IL-12 cytokine level was higher in acute hepatitis compared to non-infected subjects, while IL-2, TNF-α, and IL-10 were elevated in fulminant hepatic failure compared to acute viral hepatitis patients. Treg cells showed higher suppressive activity compared to individuals who resolved the infection [125,167]. Interestingly, the positive obese patients displayed a high inflammatory response [125]. In primary enterocytes, viral infection by different HEV strains elicited a different inflammatory response [125]. The persistence of HEV infection was recently described in immunocompromised patients [168]. In the acute phase and convalescent patients, the CD4+ and CD8+ T cells are present in peripheral blood, and their frequency declines during the months after infection resolution and is difficult to detect in chronic hepatitis patients. The chronic process is similar to other persistent viruses, such as HCV [169].

## 5. Immune Evasion Strategies

Each primary hepatotropic virus has evolved sophisticated immune evasion strategies to escape host immune responses, allowing chronic persistent infection and liver disease progression [129]. The interactions between the host, hepatitis viruses, and their elicited responses shed light on the critical role of host immunity in resolving infections or causing liver damage [170]. The host utilizes various molecules to inhibit viral replication, whereas viruses counteract these restrictions by altering molecular interactions [129]. Some proteins of hepatitis viruses play roles in the cleavage and degradation of crucial signaling molecules, interfering with them directly or indirectly to establish chronic persistent infections [156,171].

### 5.1. Immune Evasion Strategies: Hepatitis B Virus

Although, as reported above, the specific mechanisms by which HBV evades or suppresses the immune system are still unclear, the persistence of HBV is associated with viral proteins levels, particularly HBsAg and HBeAg, indicating that these proteins play a role in dampening the host immune response and evasion [172]. In HBV-infected patients, HBsAg circulates mainly as non-infectious subviral particles, such as spheres and filaments, which are present at levels 100 to 100,000 times higher than infectious virions [173]. This extensive biosynthetic effort likely serves to bind circulating anti-HBsAg antibodies, preventing them from neutralizing contagious virions in the liver [28]. Patients with chronic HBV typically lack detectable anti-HBs antibodies [174], possibly due to antibody sequestration by soluble HBsAg, the absence of HBsAg-specific B cells, or their functional impairment [174]. Several studies indicated that chronically infected patients with high HBsAg levels have a subpopulation of HBsAg-specific B cells with an atypical memory phenotype, impairing anti-HBs antibody production, despite having similar frequencies to vaccinated controls [175,176,177,178]. In contrast, HBcAg-specific B cells are more frequent in CHB patients and can mature into anti-HBc-producing cells in vitro [179,180], possibly due to lower HBcAg quantities or its ability to activate B cells independently of T cells [181]. Like observations in patients who have resolved acute hepatitis B, the loss of HBsAg in serum, associated with ‘functional’ cure, should serve as a surrogate marker for immune control and sustained suppression of viral replication. This may represent a more attainable goal than the complete eradication of HBV and could potentially allow for the discontinuation of NUCs treatment. However, recent data obtained in mouse models and from chronically infected patients contradict this issue [137,182]. Unexpectedly, the researchers revealed that the clearance of HBsAg in serum had a minimal impact on the expansion of HBV-specific naive CD8+ T cells during intrahepatic priming. It neither prevented these cells from becoming dysfunctional nor enhanced the efficacy of IL–2–based immunotherapeutic approaches in boosting their antiviral activity [182]. These findings could indicate that eliminating circulating HBsAg does not enhance HBV-specific CD8+ T cell responses in vivo, which may have significant implications for the treatment of chronic infection. On the contrary, the duration of HBsAg exposure, rather than the quantity of HBsAg, was related to the level of anti-HBV immune response [137]. These strategies to restore anti-HBV immune responses should be considered in patients younger than thirty years. Also, HBeAg, a viral protein not essential for viral assembly, replication, or infection [14], is thought to promote tolerogenic events. Mouse models show that HBeAg can act as a tolerogen for HBcAg and HBeAg-specific T cells [183,184,185] and that maternal HBeAg conditions offspring hepatic macrophages to suppress HBV-specific CD8+ T cell responses by unknown mechanisms [186]. Supporting its role in dampening T-cell responses, neonatal infections with HBeAg-negative variants often lead to viral clearance, while adult infections with same variants lead to more clinically severe disease than those with HBeAg-competent genomes [187].

### 5.2. Immune Evasion Strategies: Hepatitis D Virus

At present, it is not well-known how HDV proteins interacting with the host could determine viral evasion. However, several cellular mediators are capable of triggering innate immune responses by interaction with intracellular viral RNA [188]. Zhang et al. [189] have demonstrated that while the induction of IFN levels by HDV remains unaffected by the silencing of RIG-I or TLR3, it is almost entirely abolished following the depletion of melanoma differentiation-associated gene-5 (MDA5), the main PRRs involved in HDV recognition. Despite it showing minimal impact on HDV replication, the loss of MDA5 significantly impairs the IFN response induced by HDV. This finding highlights MDA5 as the principal sensor for detecting HDV replication intermediates, in contrast to RIG-I and TLR3. However, the precise mechanism by which MDA5 recognizes HDV RNA remains to be elucidated. Additionally, previously it was demonstrated that HDV disrupts the IFN-α-induced JAK-STAT signaling by inhibiting the STAT1, STAT2 tyrosine phosphorylation, and the receptor-associated tyrosine kinase 2 (Tyk2) [190]. This mechanism reveals how HDV evades the IFN response by targeting the JAK-STAT signaling cascade. Overall, further investigation is needed to clarify the roles of other PRRs and HDV factors in the initiation and evasion of the innate immune response. Establishing a more appropriate HBV and HDV co-infection model will be crucial for advancing this research.

### 5.3. Immune Evasion Strategies: Hepatitis C Virus

HCV strategies to evade the immune system can lead to immune cell function, leaving detrimental effects within the immune system’s memory. The nAbs produced by humoral response do not represent the driving force behind HCV infection control [163]. During chronic infection, the activation of polyclonal B cells is a typical feature. However, the E2 envelope protein, crosslinking the CD81 expressed on the cell surface, may reduce the polyclonal and monoclonal expansion of lymphocytes and inhibit the NK cell antiviral activity [130,191]. The NS proteins are involved in immune escape; HCV NS3/4A can inactivate mitochondrial antiviral-signaling protein (MAVS), downregulating RIG-I and avoiding IRF3 and NF-κB activation. The RIG-I activation is also altered by NS4B, suppressing IFN-β production [129]. Both NS3/4A and NS4B can block the immune response induced by TLR3 [192]. NS3-NS5A protease induced viral resistance through IFN signaling (IFN I and III) by blocking the IRF3/IFN I axis or activating PKR/EIF2α [130]. NS5A can inhibit MAVS-regulated signaling, binding LRPPRC protein, and can induce TLR4 expression in B cells, improving inflammatory response [193,194]. Furthermore, the inflammatory pathways are also activated by core and NS3 proteins, triggering TLR2 [191]. The core protein is also involved in the block of STAT1/STAT2 heterodimerization, inhibiting IFN signal transduction [163].

### 5.4. Immune Evasion Strategies: Hepatitis E Virus

The HEV polyproteins ORF1, ORF2, and ORF3 exhibit distinct mechanisms to evade the host immune system, crucial for understanding HEV pathogenesis and developing effective treatments. The ORF1 polyprotein of HEV plays a pivotal role in impairing the IFN I response. Nan and colleagues [195] demonstrated that the ORF1 protein inhibits type I IFN production in HEK293T cells by interfering with RIG-I signaling, achieved through the deubiquitination of both RIG-I and TBK1. This inhibition was further substantiated using a replicon system in Huh7 S10-3 cells. Additionally, the protease and methyltransferase domains of ORF1 suppress MDA5-induced IFN-β production and influence NF-kB p65 phosphorylation, with strain-dependent variability in the effect [196,197,198]. The X domain of ORF1 reduces IRF3 phosphorylation, leading to diminished IFN production, and interacts with the light chain of human ferritin, potentially affecting ferritin secretion and the innate immune response [199]. The ORF2 from HEV1–Sar55 and HEV3–Kernow directly affects IFN production by targeting IRF3 phosphorylation. It inhibits IRF3 activation through interaction with the MAVS–TBK1–IRF3 complex, a finding corroborated by co-immunoprecipitation studies in HepG2/C3A cells [200]. ORF2 also impairs TLR and RIG-I signaling pathways, though the exact molecular targets remain to be elucidated [201]. Furthermore, ORF2 disrupts apoptotic mechanisms in Huh7 cells, facilitating the completion of the viral lifecycle [202]. The ORF3 protein exhibits a complex role in regulating IFN responses, which varies by strain. It inhibits TLR3 and TLR7 expression in human THP1 and Lo2 cells, thereby reducing type I IFN production [203]. Additionally, ORF3 affects TLR3-mediated NF-κB activity in A549 cells [203] and enhances RIG-I activation and IFN-β promoter activity in HEK293T cells [204]. However, the ORF3 of HEV3–JN837481 inhibits IFN-α-induced STAT1 phosphorylation in A549 cells, which decreases ISG production [205]. This suggests a nuanced role in immune modulation, with effects on ISG15 synthesis and IFN responses varying by strain [206]. The role of ORF4 in immune evasion has yet to be investigated.

**Figure 1:**
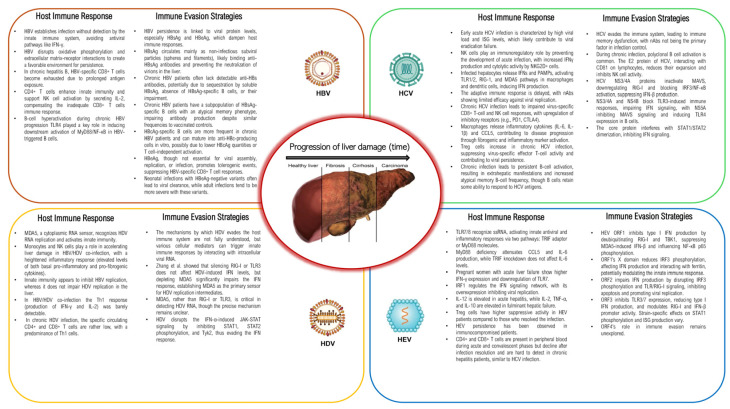
The mainly viral mechanisms involved in host immunity and immune evasion strategies. The statements included in the figure summarized the following references: [28,125,126,127,128,129,130,131,132,133,134,135,136,137,138,139,140,141,142,143,144,145,146,147,148,149,153,154,155,156,160,161,162,163,164,165,166,167,168,169,172,173,174,175,176,177,178,179,180,181,182,183,184,185,186,188,189,190,191,192,193,194,195,196,197,198,199,200,201,202,203,204,205,206].

The schematic interplay between host and viral hepatitis according to innate and adaptative immunity, with an overview of immune evasion strategies, is reported in Figure 1.

## 6. Pathogenesis and Liver Damage

The pathogenesis of viral hepatitis involves a multifaceted interplay between the virus and the host immune system, leading to liver damage. The degree of liver injury varies significantly depending on the type of hepatitis virus, the host’s immune response, and other co-factors [207]. Liver damage in viral hepatitis is primarily mediated by the host immune response rather than direct cytopathic effects of the virus. The immune system, in attempting to eliminate infected hepatocytes, can cause collateral damage to liver tissue [208]. The cytotoxic T lymphocytes (CTLs) recognize viral antigens presented on infected hepatocytes, leading to apoptosis of these cells. However, this response can also cause inflammation and necrosis, contributing to liver fibrosis and cirrhosis over time [209]. In addition to CTL-mediated damage, cytokines released by immune cells, such as interferons, tumor necrosis factor-alpha (TNF-α), and interleukins, play a significant role in the inflammatory response. These cytokines can exacerbate liver injury by promoting hepatocyte apoptosis and fibrosis through the activation of hepatic stellate cells, which produce extracellular matrix components [210]. Different hepatitis viruses employ various strategies to evade immune detection, prolonging their persistence in the host and exacerbating liver damage [211]. For instance, Hepatitis B virus (HBV) produces large quantities of subviral particles that act as decoys, distracting the immune response from targeting the actual viral particles [212,213]. Hepatitis C virus (HCV) is known for its high mutation rate, which helps it escape immune surveillance by constantly altering its antigens [214]. Hepatitis D virus (HDV), which co-infects with HBV, causes more severe liver damage due to the direct cytotoxic effect of HDV proteins [215]. Meanwhile, Hepatitis E virus (HEV), often considered a self-limiting infection, can lead to chronic hepatitis and severe liver damage in immunocompromised individuals [216]. Chronic viral hepatitis is characterized by the persistence of the virus in the liver, leading to ongoing inflammation and hepatocyte turnover [217]. The constant cycle of cell death and regeneration can result in fibrosis, a key precursor to cirrhosis [218]. As fibrosis progresses, it disrupts normal liver architecture, impairs hepatic function, and increases the risk of hepatocellular carcinoma (HCC) [219]. In chronic hepatitis B, the virus integrates into the host genome, contributing to oncogenesis. In chronic hepatitis C, the virus’s continuous replication and the resulting chronic inflammation increase the risk of HCC [220].

## 7. Current Therapies and Limitations and Emerging Therapeutic Strategies

Viral hepatitis, including hepatitis A, B, C, D, and E, poses a significant global public health challenge. Each type of hepatitis has unique characteristics in terms of epidemiology, modes of transmission, and disease progression, necessitating specific therapeutic approaches. Current therapies primarily focus on managing infection and its complications, yet they are not without limitations. Fortunately, emerging therapeutic strategies offer hope for more effective and targeted treatments (see Table 3).

For hepatitis A, an acute infection transmitted through the fecal-oral route, the primary form of prevention is vaccination, which has been highly effective in reducing incidence rates in areas with high vaccination coverage [221]. However, treatment options for those infected are limited to supportive care, as the disease is usually self-limiting and does not lead to chronic infection [222]. The main limitation here is the lack of a specific antiviral therapy that can accelerate recovery or prevent severe cases, which, though rare, can occur, particularly in older adults or individuals with underlying health conditions.

Hepatitis B, a chronic infection that can lead to serious complications such as cirrhosis and hepatocellular carcinoma, has more extensive therapeutic options. Current treatments include nucleos(t)ide analogs like tenofovir and entecavir, which effectively suppress viral replication and reduce the risk of liver damage. Additionally, pegylated interferon-alpha is used in some cases to enhance the immune response. Despite the effectiveness of these therapies in managing the disease, they do not cure the infection; the virus can persist in the liver in a dormant state, leading to the possibility of reactivation. This necessitates lifelong treatment for many patients, with associated costs and potential side effects. Furthermore, not all patients respond adequately to current therapies, and drug resistance can develop, underscoring the need for novel therapeutic approaches [223].

In parallel with therapeutic interventions, the hepatitis B vaccine represents one of the most significant advancements in preventing viral hepatitis and its associated complications. The hepatitis B vaccine, developed through recombinant DNA technology, consists of HBsAg and is highly effective in stimulating a protective immune response. Vaccination induces the production of antibodies against HBsAg, which neutralize the virus and prevent infection. The vaccine is administered in a series of three doses at 0, 1, and 6 months, although alternative schedules, including a four-dose regimen, are used in certain high-risk populations or in infants born to HBsAg-positive mothers to enhance early protection. The efficacy of the hepatitis B vaccine is well documented, with protection rates above 95% in healthy individuals who complete the full vaccine course [224]. This high level of efficacy has led to widespread adoption of the vaccine in national immunization programs around the world, significantly reducing the incidence of new infections. For example, countries that have implemented universal vaccination of infants have seen a dramatic decrease in the prevalence of hepatitis B, particularly in younger age groups [225]. In Taiwan, for example, the introduction of the hepatitis B vaccination program in the 1980s led to a reduction in rates of chronic hepatitis B infection among children from about 10% to less than 1% [226]. In addition to individual protection, the hepatitis B vaccine also contributes to herd immunity, which is essential to controlling the spread of the virus in populations. Herd immunity provides indirect protection to unvaccinated individuals, including those who are immunocompromised or have contraindications to the vaccine. In addition, vaccination significantly reduces the risk of hepatitis B-related complications, such as cirrhosis and hepatocellular carcinoma, by preventing chronic infection [227].

The safety profile of the hepatitis B vaccine is also robust: most adverse events are mild and transient, such as injection site pain or low-grade fever. Serious adverse events are extremely rare, and the benefits of vaccination far outweigh the risks [228]. In addition, the durability of the vaccine is impressive: most vaccinated individuals maintain protective antibody levels for decades, and booster doses are generally not necessary in immunocompetent individuals [229].

Hepatitis C has seen remarkable advancements in treatment with the introduction of direct-acting antivirals (DAAs). These drugs target specific steps in the viral replication cycle, offering a highly effective and often curative treatment option. DAAs have transformed the management of hepatitis C, with cure rates exceeding 95% in many cases and a much shorter duration of therapy compared to older treatments like interferon and ribavirin. However, challenges remain, including the high cost of these medications, which limits access in many low- and middle-income countries. Moreover, while DAAs are highly effective at eliminating the virus, they do not protect against reinfection, a significant concern in populations with ongoing risk factors, such as people who inject drugs [230].

Hepatitis D, which only occurs in conjunction with hepatitis B, presents a particularly challenging therapeutic scenario. Current treatments are limited, with pegylated interferon-alpha being the most commonly used option. However, this treatment has modest efficacy and is associated with significant side effects, leading to poor tolerability in many patients. The development of new therapies, such as entry inhibitors like bulevirtide, offers a promising direction for the future. Bulevirtide has shown efficacy in reducing viral load and improving liver function, but its long-term benefits and safety profile require further study [230,231].

Hepatitis E, primarily transmitted through contaminated water and food, is generally a self-limiting infection. However, in immunocompromised individuals and pregnant women, it can lead to severe complications. Currently, there is no specific antiviral treatment for hepatitis E; management focuses on supportive care. Ribavirin has been used off-label with some success in chronic cases, particularly in immunocompromised patients. The development of a hepatitis E vaccine represents a significant step forward, but it is not yet widely available, and research into specific antiviral therapies remains limited [232].

The limitations of current therapies across all types of hepatitis highlight the need for ongoing research and development of new treatment options. Emerging therapeutic strategies are increasingly focusing on targeting the viral life cycle more precisely, modulating the immune response, and addressing the specific needs of diverse patient populations. For instance, novel antivirals that can cure hepatitis B or inhibit hepatitis D co-infection are under investigation, as are immunomodulatory therapies that aim to enhance the body’s natural ability to clear the virus [231].

Gene editing technologies, such as CRISPR-Cas9, are also being explored as potential tools for directly targeting and eradicating viral genomes within infected cells. Such approaches could offer the possibility of a functional cure, particularly for hepatitis B, by eliminating the viral reservoir. Additionally, therapeutic vaccines that could boost the immune response against chronic hepatitis infections are in various stages of development and could complement existing therapies [233].

In general, while significant progress has been made in the treatment of viral hepatitis, current therapies have notable limitations, including the need for lifelong treatment, potential side effects, and limited efficacy in certain cases. The advent of emerging therapeutic strategies, including new antivirals, immunomodulators, gene editing technologies, and therapeutic vaccines, holds promise for more effective and possibly curative treatments. Continued research and equitable access to these innovations will be crucial in the global effort to control and ultimately eliminate viral hepatitis as a public health threat.

**Table 3:** Summary of main antiviral targets for different hepatitis viruses.

Virus	Antiviral Targets	Mechanism of Action	Example of Therapeutics	References
HAV	RNA-dependent RNA polymerase	Inhibits viral RNA synthesis	No specific approved antiviral therapy; supportive care	[234]
	Immune modulation	Enhances host immune response	HAV vaccination (preventive), immune globulin	[235,236]
HBV	Reverse Transcriptase	Inhibits DNA replication	Tenofovir, Entecavir, Lamivudine	[237,238]
	HBsAg	Inhibits viral entry and immune evasion	Vaccination (preventive), pegylated interferon	[239,240]
HCV	NS3/4A Protease	Inhibits viral polyprotein processing	Simeprevir, Grazoprevir	[241,242]
	NS5B RNA-dependent RNA polymerase	Blocks viral RNA replication	Sofosbuvir	[243,244]
	NS5A Protein	Disrupts viral replication and assembly	Ledipasvir, Velpatasvir	[245,246]
HDV	HDAg	Inhibits viral replication	Pegylated interferon, Bulevirtide (HDV entry inhibitor)	[247,248]
	HBsAg (in HBV/HDV co-infection)	Prevents HDV assembly and release	HBV vaccination (indirect protection against HDV)	[249]
HEV	RNA-dependent RNA polymerase	Inhibits viral RNA synthesis	Ribavirin (off-label use in severe cases)	[250]
	Immune Modulation	Enhances host immune response	No specific approved antiviral therapy; supportive care	[251]

## 8. Conclusions

In a context where viral hepatitis research has already achieved significant milestones, significant gaps remain in the in-depth understanding of virus–host interactions, especially with regard to mechanisms of immune evasion and viral persistence. The purpose of this review was to fill these gaps by offering a critical synthesis of the most recent findings, with a focus on the less explored mechanisms that modulate immune responses by hepatitis viruses. To ensure a thorough analysis and maximize the impact of this review, we decided to focus on a few key aspects of viral hepatitis. In particular, the review focused on the immune evasion mechanisms adopted by hepatitis viruses and their implications for disease progression and the development of new therapeutic strategies. This selective approach allowed us to avoid an overly broad treatment that might have reduced the effectiveness of the analysis and instead offer a more detailed and critical exploration of these central themes, thus providing the reader with a deeper understanding of the challenges and opportunities present in the field.

To guarantee a rigorous and representative analysis of the state of the art regarding host–pathogen interaction in viral hepatitis, we followed a highly structured literature selection process. The search was conducted through relevant scientific databases such as PubMed, Scopus, and Web of Science, using targeted keywords such as ‘viral hepatitis’, ‘immune evasion’, ‘host–pathogen interaction’, and ‘therapeutic strategies’. Only peer-reviewed studies published within the last 10 years were included, with an emphasis on articles presenting robust and innovative empirical data. Older studies were considered only if they were fundamental to the understanding of key concepts or provided a necessary historical basis for the context of the review. Studies were selected on the basis of specific criteria: only those with a high level of evidence, such as randomized controlled trials, meta-analyses, and systematic reviews, were included, while articles based on expert opinions or data not supported by empirical evidence were excluded. This approach minimized the risk of bias and provided a balanced and detailed view of the interactions between hepatitis virus and the host immune system.

In addition to providing a comprehensive synthesis of the existing literature, this review took a critical approach, analyzing the various therapeutic strategies and scientific evidence available in the field of viral hepatitis. Specifically, we did not simply summarize the data but critically evaluated the effectiveness of current therapies, comparing their successes and failures and identifying areas where further research is needed. This approach allowed us to highlight not only existing gaps but also potential future directions for research and development of new therapies, with a focus on emerging strategies such as gene editing technologies and therapeutic vaccines.

While we have adopted a rigorous literature selection process, we recognize that there are some inherent limitations. In particular, most of the available studies focus on specific populations or animal models, which could limit the generalizability of the results to different populations. In addition, sample quality and size varied among studies, potentially affecting the robustness of the conclusions drawn. We sought to address these limitations by critically discussing the results according to the methodological characteristics of each study to provide a balanced presentation of the available evidence.

In conclusion, viral hepatitis continues to be a major global health concern, significantly contributing to liver disease morbidity and mortality. The ability of hepatitis viruses, particularly hepatitis B, C, D, and E, to manipulate host metabolic and signaling pathways to facilitate their own survival and replication underscores the sophistication of these pathogens. Moreover, the host’s innate and adaptive immune responses play critical roles in attempting to control and eliminate viral infections, yet these responses can also contribute to liver damage when dysregulated. A critical insight from this review is the role of genetic, epigenetic, and environmental factors in influencing individual susceptibility to viral hepatitis and its progression. These factors not only affect the severity of the initial infection but also determine the likelihood of chronic disease development, underscoring the importance of personalized approaches in managing viral hepatitis. The persistence of these viruses in the host, leading to chronic infection and potential progression to cirrhosis and hepatocellular carcinoma, highlights the urgent need for effective therapeutic strategies. The exploration of viral persistence mechanisms offers promising avenues for novel treatments, as fully understanding these processes is pivotal for the development of interventions that can disrupt viral life cycles and enhance host immune clearance. This review also emphasizes the potential of targeting specific host–virus interactions as a therapeutic strategy, which may lead to the discovery of new drug candidates capable of preventing or reversing chronic infections.

In light of these findings, future research should focus on unraveling the remaining mysteries of hepatitis virus biology and host interactions, as well as the development of innovative therapeutic approaches that can mitigate the burden of viral hepatitis. The pursuit of a comprehensive understanding of these complex host–pathogen dynamics not only holds the promise of improving patient outcomes but also contributes to the broader goal of controlling and eventually eradicating viral hepatitis as a public health threat.

## Figures and Tables

**Table 1:** Summary of hepatitis viruses virological and pathogenetic characteristics, including therapeutic approach, discussed through the review.

Major Points	Content of Discussion
Overview of Viral Hepatitis and Host–Pathogen interactions	Viral hepatitis primarily affects the hepatic cells. The host–pathogen interaction is pivotal in determining disease progression and outcomes across the spectrum of hepatitis viruses.
Mechanisms of Viral Entry	The entry of hepatotropic viruses into liver cells relies on specific molecular mechanisms unique to each virus. The viral entry started the progression of liver disease in acute or chronic hepatitis.
Innate and Adaptive Immune Response	The innate immune system quickly detects viruses through receptors like TLRs and induces interferons to activate antiviral genes. Adaptive immunity, primarily through T and B cells, helps resolve acute infection or contributes to viral persistence.
Immune Evasion Strategies	Hepatotropic viruses could manipulate host immune responses by degrading key signaling molecules, determining infection resolution or chronicity.
Pathogenesis and Liver Damage	The pathogenesis of viral hepatitis results from complex interactions between the virus and the host immune system, causing different degrees of liver damage.
Current and Emerging Therapeutic Strategies	Even if current therapies can manage viral hepatitis and its complications, emerging treatments hold promise for more effective and targeted solutions.

**Table 2:** Schematic summary of epidemiological, virological, and clinical aspects of primary hepatotropic viruses.

Type of Virus Characteristics	HAV	HBV	HCV	HDV	HEV
Family	*Picornaviridae*	*Hepadnaviridae*	*Flaviviridae*	*Deltaviridae*	*Hepeviridae*
Genus	*Hepatovirus*	*Orthohepadnavirus*	*Hepacivirus*	*Deltavirus*	*Orthohepevirus*
Genome Characteristics	Quasi-enveloped, ssRNA, ~7.5 kb	Enveloped, partial dsDNA, ~3.2 kb	Enveloped, ssRNA, ~9.6 kb	Enveloped, ssRNA, ~1.7 kb (requires HBV for replication)	Quasi-enveloped, ssRNA, ~7.2 kb
First Discovery (year)	1973	1965	1989	1977	1983
Prevalence	1.4–1.5 million annually	350 million chronically infected	58 million chronically infected	10–20 million co-infected with HBV	20 million cases annually
Incidence	1.4 million new cases annually	10 million new cases annually	1.5 million new cases annually	500,000 new cases annually	3.3 million symptomatic cases annually
Transmission Route	Fecal-oral	Blood, sexual contact, perinatal	Blood, intravenous drug use, sexual contact	Blood, sexual contact, perinatal (requires HBV co-infection)	Fecal-oral, zoonotic (genotypes 3, 4), from mother to child
Type of hepatitis	Acute	Acute, chronic	Acute, chronic	Acute, chronic (with HBV)	Acute, chronic
Clinical symptoms	Jaundice, nausea, abdominal pain, fatigue, loss of appetite	Jaundice, fatigue, liver cirrhosis, HCC	Fatigue, jaundice, liver cirrhosis, HCC	Exacerbation of HBV symptoms, rapid progression to cirrhosis, HCC	Jaundice, fatigue, abdominal pain, more severe in pregnant women
Diagnosis	Serology (IgM anti-HAV), PCR	Serology (HBsAg, anti-HBc, HBeAg), PCR	Serology (anti-HCV), PCR	Serology (anti-HDV), PCR	Serology (IgM anti-HEV), PCR
Treatment	Supportive care	Nucleos(t)ide analogues, interferon	Direct-acting antivirals	Pegylated interferon-alpha, entry inhibitor Bulevirtide (authorized in Europe in July 2020)	ribavirin or pegylated interferon-α
Prevention	Vaccination, improved sanitation	Vaccination, safe sex practices, blood screening	Blood screening, safe injection practices	HBV vaccination, safe sex practices	Improved sanitation, vaccine available in China

Note: Global prevalence and incidence are available in the latest WHO reports as of 2024 [1]. The treatment and prevention methods are based on current medical guidelines and practices [15].
